# Nano-Tubular Cellulose for Bioprocess Technology Development

**DOI:** 10.1371/journal.pone.0034350

**Published:** 2012-04-09

**Authors:** Athanasios A. Koutinas, Vasilios Sypsas, Panagiotis Kandylis, Andreas Michelis, Argyro Bekatorou, Yiannis Kourkoutas, Christos Kordulis, Alexis Lycourghiotis, Ibrahim M. Banat, Poonam Nigam, Roger Marchant, Myrsini Giannouli, Panagiotis Yianoulis

**Affiliations:** 1 Food Biotechnology Group, Department of Chemistry, University of Patras, Patras, Greece; 2 Department of Molecular Biology, Democritus University of Thrace, Alexandroupolis, Greece; 3 Group of Catalysis and Interfacial Chemistry for Environmental Applications, University of Patras, Patras, Greece; 4 Institute of Chemical Engineering and High Temperature Chemical Processes (FORTH/ICE-HT), Patras, Greece; 5 School of Biomedical Sciences, University of Ulster, Coleraine, United Kingdom; 6 Department of Physics, University of Patras, Patras, Greece; RMIT University, Australia

## Abstract

Delignified cellulosic material has shown a significant promotional effect on the alcoholic fermentation as yeast immobilization support. However, its potential for further biotechnological development is unexploited. This study reports the characterization of this tubular/porous cellulosic material, which was done by SEM, porosimetry and X-ray powder diffractometry. The results showed that the structure of nano-tubular cellulose (NC) justifies its suitability for use in “cold pasteurization” processes and its promoting activity in bioprocessing (fermentation). The last was explained by a glucose pump theory. Also, it was demonstrated that crystallization of viscous invert sugar solutions during freeze drying could not be otherwise achieved unless NC was present. This effect as well as the feasibility of extremely low temperature fermentation are due to reduction of the activation energy, and have facilitated the development of technologies such as wine fermentations at home scale (in a domestic refrigerator). Moreover, NC may lead to new perspectives in research such as the development of new composites, templates for cylindrical nano-particles, etc.

## Introduction

Research on bioprocess technology development has been extensive during the last decades; however most of these technologies have not been used at industrial level. The main problems associated with these technologies are related to productivity, ease of industrial application and production cost. However there are still opportunities to use abundant, low cost materials with specific chemical or nano-mechanical properties to create multiple new, effective bioprocessing systems.

One of the major problems of food and drug production is the use of the relatively costly pasteurization processes. In addition to high operational cost, pasteurization reduces the nutritional quality of food products. However due to its importance, pasteurization is extensively used despite these disadvantages. Only in some food bioprocesses like wine production, membranes [1] are used to remove e.g. *Leuconostoc oenos* cells but they have a high cost and are not easy to handle at industrial level. Alternatively, if a nano-tubular solid of food grade purity, could be used to remove microbial cells through cell entrapment and immobilization at ambient and low temperatures, it could have wide applications in the food and pharmaceutical industries, such as the cold pasteurization of liquid foods and the cold sterilization of liquids that are used in drug production plants, respectively.

Porous materials like γ-alumina pellets [2] and mineral kissiris [3] have been used for cell entrapment and immobilization; however their composition (that liberates aluminium in the processed media) limits their applications. Especially their use in food and drug production is not recommended. On the other hand delignified cellulosic material is of food grade purity, abundant in nature and therefore of low cost. This material has been successfully used as support for cell immobilization [4]. The produced biocatalyst was used for extremely low temperature fermentations in order to improve the quality of the products [5], and it was found able to promote the alcoholic fermentation of molasses [6].

Nanotechnology is one of the most growing areas of research the last decades. Especially the subarea of nano-cellulosic materials has attracted considerably high-level attention from researchers. The applications of this technology are numerous and in many fields. For example cellulosic nano-fibrils coated with SnO_2_ have been used for the production of membranes [7], while nano-porous cellulose combined with carbon nano-tubes have been used in batteries and supercapacitors [8].

Following that trend the main goal of the present work was to use nano-tubular cellulose (NC) for the development of bioprocesses related to food industries. More specifically the work addresses the characterization of the tubular structure of NC and its suitability, due to that structure, for (i) “cold pasteurization” processes, (ii) promotion of alcoholic fermentation, (iii) extremely low temperature fermentations, and (iv) home-scale (domestic refrigerator) bioprocesses. Finally the perspectives for NC applications in nanobiotechnology are discussed, taking into account the results of the present study.

## Results and Discussion

### Nanostructure and microstructure of tubular cellulose

**Figure 1:**
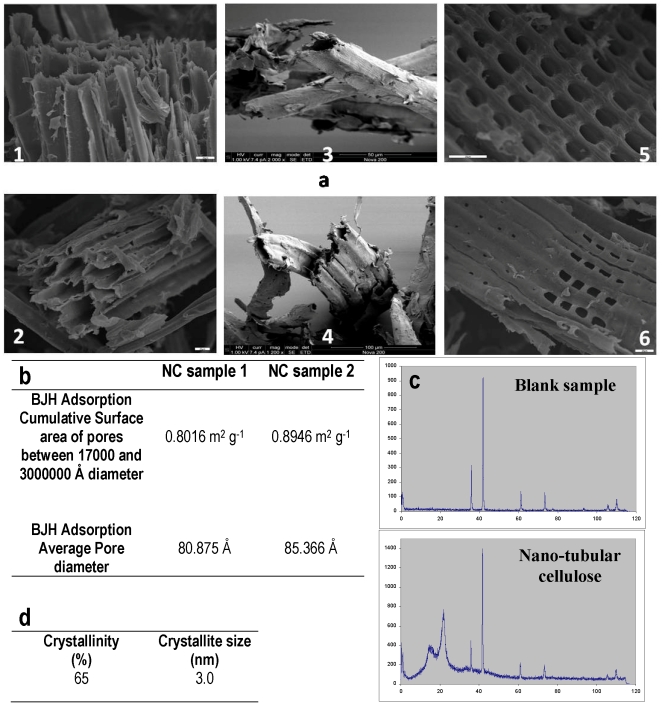
Characterization of nano-tubular cellulose (NC). (a) SEM micrographs of NC produced by delignification of sawdust with NaOH (scale bar in 1, 2, 5, 6 corresponds to 20 µm, while in 3 to 50 µm and in 4 to 100 µm). (b) NC average pore diameter and cumulative surface area of the pores. (c) Spectra from X-ray diffractometry of blank sample and NC sample. (d) Crystallinity and crystallite size of NC calculated using spectra from Figure 2C.

In the context of limited availability of complex materials for advanced bioprocessing, this investigation initially examines (i) the characterization of the structure of the NC material, (ii) the crystallinity of NC, and (iii) the degree of its crystallinity. Figure 1A shows the tubular structure in the remaining mass of cellulose after removal of lignin, while Figure 1B illustrates that the tubes have nano-scale dimensions. The cumulative surface area of the tubes is in the range of 0.8–0.89 m^2^ g^−1^ as indicated by porosimetry analysis. This surface is relatively small compared with other porous materials such as γ-alumina [9]. However, using a natural organic tubular biopolymer is attractive from the point of view that it is safer for bioprocess applications. The relatively small surface can be attributed to the fact that the lignin content in sawdust is about 16% and therefore its removal leads to limited tubing in the remaining mass of cellulose. However, this tubing is enough to encourage application for bioprocess development. Furthermore, single tubes (Fig. 1A, panel 3) and small groups of 3–4 tubes (Fig. 1A, panel 4) are observed. The tubes are horizontal (Fig. 1A, panel 2) and vertical (Fig. 1A, panel 1) and contain holes of varying diameters covering a relatively small area of the tubes (Fig. 1A, panel 6). Tubes with big differences of diameter are shown in the SEM photo of Figure 1A, panel 1. Likewise, tubes forming normal patterns such as a normal square are shown in Figure 1A, panel 5. However, Figure 1A, panel 1, 1A, panel 2 and 1A, panel 6 show tubes of small and bigger diameters. In the SEM micrographs it is shown that the bigger tubes are in micro-scale dimension and the small ones in nano-scale. Therefore, there is a mixture of micro and nano-tubing. The cumulative surface area and average pore diameter of the nano-tubes was measured by porosimetry. Figure 1D illustrates that NC has 65% degree of crystallinity indicating that the treatment with sodium hydroxide applied for delignification did not destroy its crystal structure. The size of the crystallites is 3 nm, which is big enough to contain a part of the tubes. Therefore, NC provides the possibility of cell entrapment and immobilization. These three different potential properties of NC make this nano-material suitable for different bioprocesses development as described above. Furthermore, this natural structure facilitates various new perspectives in research, which are discussed in this paper and which point to new nano-structured materials development. NC was produced by delignification of softwood sawdust and the observed microstructures reflect the architecture of the tracheids in the wood. The use of other starting materials would produce slightly different end products. In any case it is necessary to increase the nano-tubing and microtubing of cellulose. Pure microbial cellulose containing higher percentages of lignin could be examined, that would lead to more extended tubing after lignin removal.

### Cold pasteurization of liquid foods

**Figure 2:**
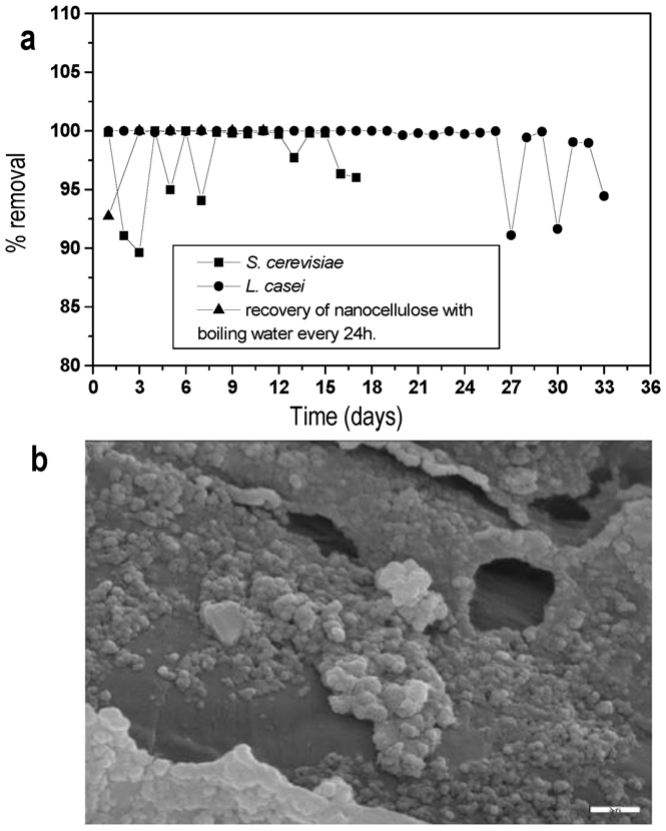
Nano-tubular cellulose (NC) in food, environmental and health care applications. (a) Removal of bacteria and yeasts from water (22–26°C). (b) SEM micrograph of *L. casei* flocculation in NC that was sampled from the bioreactor, which was pumped at low temperature (5°C) with its liquid culture for cold pasteurization (scale bar corresponds to 10 µm).

NC can be used to remove microorganisms from liquid foods or liquids used in the pharmaceutical industries at ambient and low temperatures. The aim is to develop a novel technology for cold pasteurization of drinking water, fruit juices, milk, wine, beer and other beverages, which could also be applied in pharmaceutical processing liquids. To test this possibility, NC was selected as a suitable new material due to its nano-tubular structure, ideal for the entrapment and immobilization of cells [4]. Water contaminated with bacteria and yeasts was passed continuously through the nano-tubular NC filter and the filtrate was analyzed for viable cells. The experiments were carried out at ambient temperature. There was an obvious difference in turbidity among the contaminated influent and the effluent water. Figure 2A shows that the microbiological load removal was 100%. The operational stability at this level of microbiological load removal remained constant for two days and then dropped. It was restored to 100% after regeneration of the NC filter achieved by washing it with hot water. The regeneration was successfully repeated every two days for more than one month, keeping the microbial removal at 100%. After the success of this experiment, cold pasteurization of the contaminated flow was carried out at 3–5°C since food processing is preferred at low temperatures to maintain the nutritional value and quality of the food. However, the removal of cells was greatly reduced and the effluent water was turbid. This unsuccessful result was attributed to the crystallization of sodium chloride added to the water to maintain the osmotic balance of cells. The crystals seemed to block the NC tubes preventing cell entrapment. Additionally, cell flocculation caused by low temperature stress [10], could also block the tubes. Figure 2B illustrates the formation of microbial flocks and proves that the reduction in microbial removal can be attributed to cell flocculation.

Although low temperature cold pasteurization of the model system (contaminated water) failed, the experimental work involving a real food, i.e. orange juice, was attempted. Cold pasteurization of orange juice should be performed at low temperatures to preserve the quality characteristics, and it could be presumed successful since components in the juice (like ascorbic acid) are essential for the synthesis of substances like carnitine that reduce the stress of cells [11]. Indeed, the low temperature cold pasteurization of orange juice resulted in 100% microbial load removal, as demonstrated by plate counting of influent and effluent samples (data not shown).

### Nano-tubular cellulose as promoted of the alcoholic fermentation

**Figure 3:**
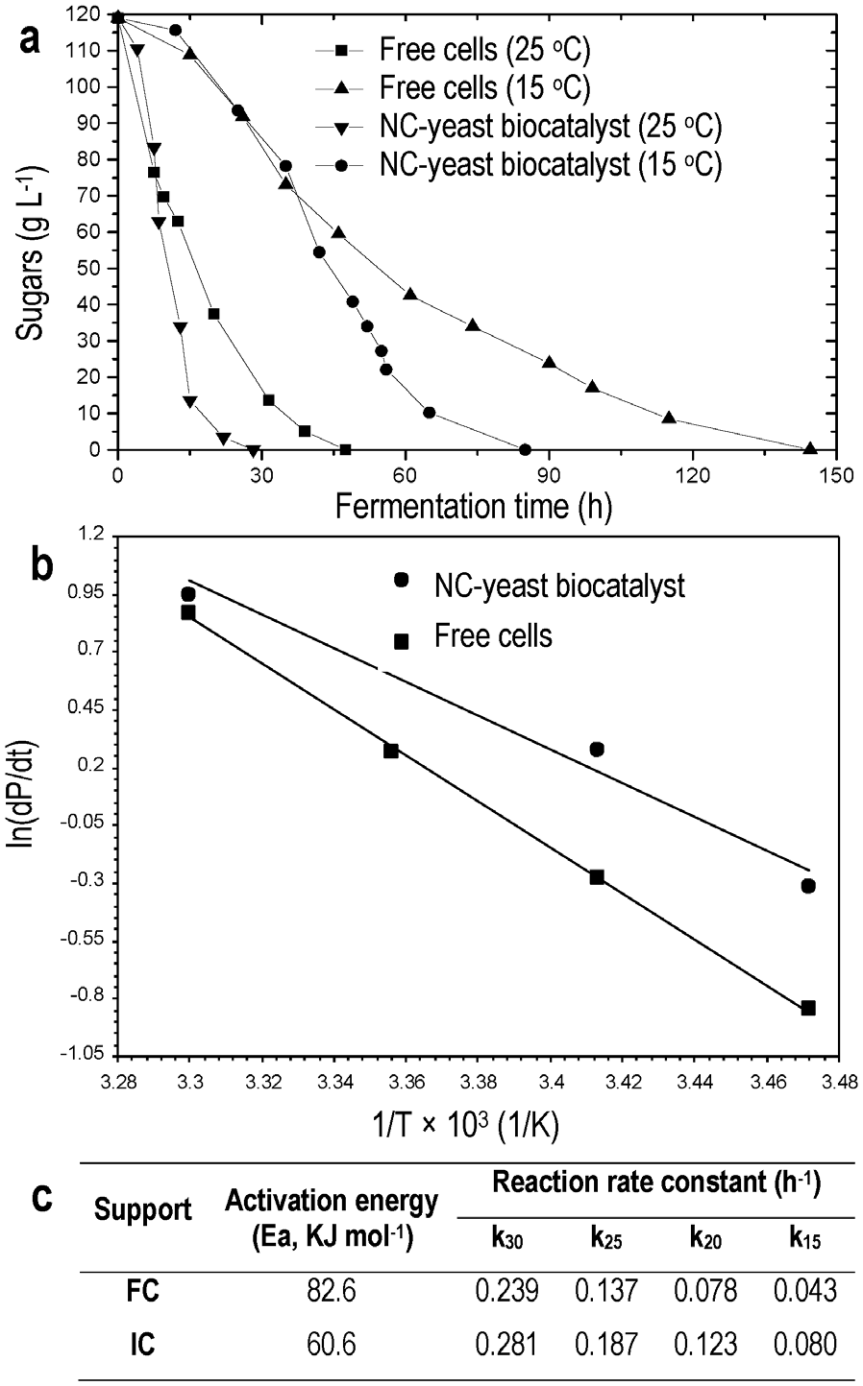
Nano-tubular cellulose (NC) and promotion of the alcoholic fermentation. (a) Fermentation kinetics observed at 25°C and 15°C by free cells and cells immobilised on NC (NC-yeast biocatalyst). (b) Arrhenius plot for evaluation of the activation energy and the pre-exponential factor of alcoholic fermentation performed with free and NC-yeast biocatalyst. (c) Activation energies and reaction rate constants of the fermentations made using free cells (FC) and cells immobilised on NC (IC).

Enhancement of the alcoholic fermentation of molasses by delignified cellulosic material has been exhibited [6]. This observation motivated further research to examine the physicochemical structure of delignified cellulosic material and correlate it with its promotional effect on the rate of alcoholic fermentation. The characterization showed that cellulose has nano-scale tubular pores. This nano-tubular material (NC) was used as yeast immobilisation support to examine the activation energy E_a_ of the alcoholic fermentation and the reaction speed constant k versus temperature, in comparison with free yeast cells, in order to prove if NC affects the catalytic activity and therefore acts as promoter of the alcoholic fermentation. Fermentations were performed at different temperatures in the presence of NC and with free cells, separately. Figure 3A shows that the NC biocatalyst increased the fermentation rate and was more effective as the temperature was reduced compared with free cells. Furthermore, Figure 3B and 3C shows that the activation energy E_a_ of the NC biocatalyst was 28% lower than that of free cells. Likewise, the reaction speed constant k was higher for the NC biocatalyst. These results indicate that NC is an excellent carrier to promote the catalytic action of cells for the fermentation of molasses, as it was observed for delignified cellulosic material by Iconomou et al. [6]. It seems that this catalytic behaviour of NC is strongly related to its tubular structure, and can find application in fuel-grade ethanol production by increasing productivity and allowing a reduction in the size of the production plant [12].

NC is the first natural nano-tubular material characterized as a promoter of a bioconversion and the first biomolecule that promotes the alcoholic fermentation. The effect on the catalytic activity by NC that contains functional active hydroxyl groups is in agreement with a recent study, which proposed that effective catalysts can be created by adding functional hydroxyl groups in materials with nano-tubes, for example carbon nano-tubes [13]. This creates a new opportunity in research to examine this material as a possible catalytic promoter of other biochemical reactions like the reaction of lactic acid fermentation, malolactic fermentation, or the oxidation of alcohol by acetic acid bacteria to acetic acid. Also, its chemical and physical structure that has been studied in the context of this investigation can provide the base for the synthesis of new promoters of bioprocesses, having similar polymeric structures.

### Glucose pump theory for the promotion of fermentation by nano-tubular cellulose

**Figure 4:**
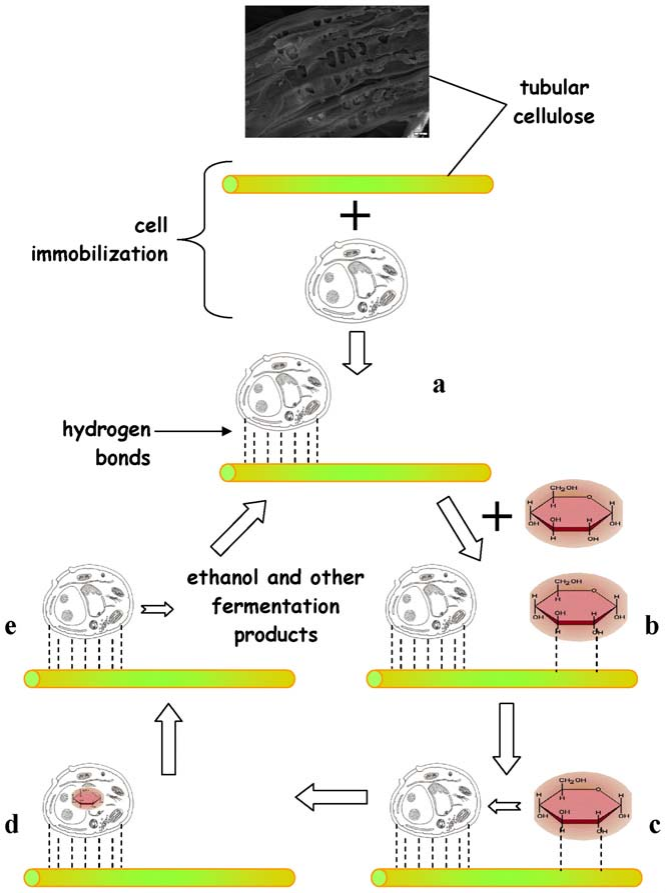
“Glucose pump”. (a) Cell is immobilized on cellulose fiber by hydrogen bonding. (b) Glucose also attached on the surface of the cellulose fiber by hydrogen bonding. (c) Glucose is transferred inside the cell. (d) Glucose is biochemically converted inside the cell. (e) After glycolysis, alcoholic fermentation and other processes, ethanol and other fermentation products are produced and transferred to the solution, and another cycle begins.

The catalytic effect of NC on the fermentation rate can be attributed to a glucose pump operating among the system of cellulose tubes that increase the surface, cells and glucose. The operation and rationale of a glucose pump is presented in Figure 4. According to this theory cells are encapsulated and joined by hydrogen bonding with hydroxyl groups between the surface of NC and the cell wall. Likewise, glucose molecules are joined with cellulose at the surface of the tubes through their hydroxyl group hydrogen bonds. Therefore, high cell and glucose concentrations are created mainly on the surface of the cellulose nano-tubes, increasing the rate of the biochemical reaction. Another factor that may affect the rate of the reaction is that glucose molecules and cells that are attached on the surface of the nano-tubes come very close to each other, increasing the glucose uptake rate. The bioconversion of glucose by the cells liberates alcohol, leading to continuous glucose pumping from the solution, mainly to the surface of the nano-tubes.

### Nano-tubular cellulose for domestic refrigerator bioprocess development

**Figure 5:**
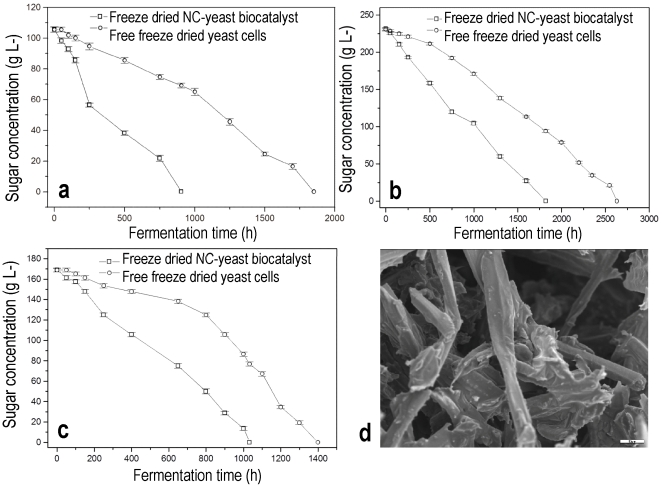
Domestic refrigerator wine making. (a) Fermentation kinetics observed at 1°C of freeze dried mixture of invert sugar and NC-yeast biocatalyst. (b) Fermentation kinetics at 1°C of freeze dried mixture of grape must and NC-yeast biocatalyst. (c) Fermentation kinetics observed at 1°C of freeze dried mixture of raisins and NC-yeast biocatalyst. Fermentation kinetics using free freeze dried cells are also shown in all Figures (a), (b) and (c). (d) SEM micrograph of the freeze dried mixture of grape must and NC-yeast biocatalyst (scale bar corresponds to 20 µm).

The catalytic effect and reduction of the activation energy Ea of the alcoholic fermentation by NC, explains the high increase of the fermentation rate at low temperatures that was observed when delignified cellulosic materials where used as yeast immobilisation supports [4]. The feasibility of low temperature fermentation led to the idea of developing technologies for producing foods at home scale. Domestic wine making was selected to show the possibility for the development of bioprocesses that the consumers could apply in their own refrigerators. To develop this technology using NC, the strategy adopted was the preparation of a powder that could be preserved for a long period and consists of freeze dried nano-tubular cellulose supported biocatalyst and solidified grape must. The process is described in detail in the Methods section. The problem was that after freeze drying of grape must, the product was a very viscous liquid that could not be solidified by further freeze drying. However, carrying out freeze drying of grape must in the presence of NC a crystalline solid mass was formed containing crystals of glucose and fructose. Figure 5D shows an electron micrograph of this solid product with crystals of sugars derived from the viscous liquid grape must. The crystals are formed inside a nano-compartment. Crystal formation in the presence of NC may be explained by considering that some of the adsorption sites for the sugar molecules inside the tubes may act as crystallization centres promoting interfacial heterogeneous crystallization [14]. Another observation is that cells encapsulated in the NC tubes were not destroyed and remained alive, although they were in a very viscous solution and therefore under increased osmotic stress. This protection of freeze dried cells attached in the tubes of NC is attributed to the water content of the viscous sugar solution, which, even though low, forms hydrogen bonds in the tubes with the hydroscopic cellulose macromolecules and is retained by it. In addition, the fact that crystallization of the sugars did not damage the cell walls of the yeast cells may be due to the protective effect of dehydration by freeze-drying.

Figure 5A shows the kinetics of the model fermentation system using commercial invert sugar powder. The NC-yeast biocatalyst resulted in a dramatic reduction of the fermentation time as compared with free cells. The results of the model fermentation provided a basis for the second experiment using powdered grape must, the results of which are presented in Figure 5B. These results show that both fermentations, which were carried out at 1°C, were completed and the NC resulted in an increase of the fermentation rate and reduction of the fermentation time in comparison with free cells. As an alternative strategy in the development of the technology, dried grapes were used to substitute solid freeze dried grape must. Figure 5C shows that once again there was a reduction in the fermentation time in the case of the NC biocatalyst.

**Figure 6:**
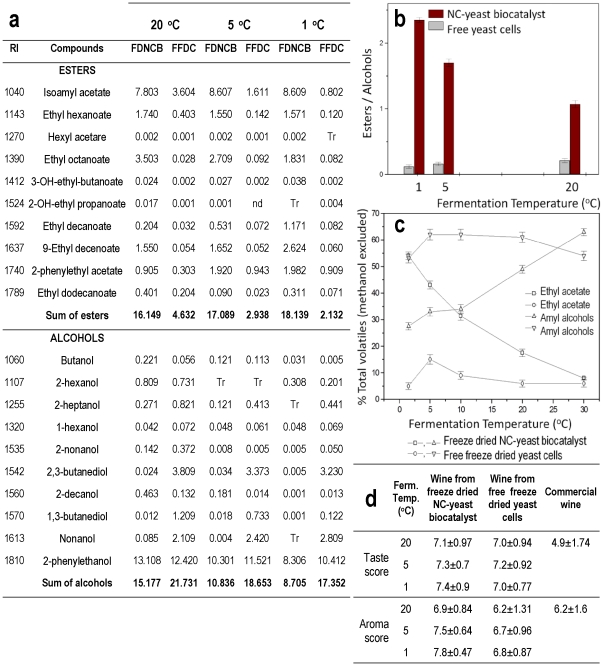
Quality of the wine produced in the refrigerator using mixture of freeze dried grape must and freeze dried biocatalysts. (a) Effect of temperature on esters and alcohols formation during alcoholic fermentation of mixtures of freeze dried grape must and freeze dried biocatalysts. Tr: compounds <1 µg/L (traces), nd: not detected. FDNCB: Freeze dried NC-yeast biocatalyst, FFDC: Free freeze dried cells. (b) Effect of temperature on the ratio of esters-to-alcohols formed during alcoholic fermentation of mixtures of freeze dried grape must and freeze dried biocatalysts. (c) Effect of temperature on the (%) percentage of ethyl acetate and amyl alcohols on total volatiles formed during alcoholic fermentation of mixtures of freeze dried grape must and freeze dried biocatalysts. Plots show means of three replicates with standard errors. (d) Sensory evaluation of commercial wine and wines produced by freeze dried free cells and freeze dried NC-yeast biocatalyst.

Using NC, the improvement of wine quality is shown by the increase of esters and reduction of higher-alcohols as compared with free cells (Figure 6A). Moreover, Figure 6B and 6C reveals the reduction of amyl alcohols and increase of ethyl acetate on total volatiles as the temperature was reduced. These results lead to improvement of wine quality that has also demonstrated by sensory testing (Fig. 6D).

### Nano-tubular cellulose for creation of new trends in research

NC may be tested (a) as template for the production of cylindrical nano-particles [15]. The precipitation or gel formation inside the tubular pores may be followed by thermal treatment to stabilize the fibres whereas the removal of the template could be obtained by combustion or hydrolysis of cellulose using cellulases [16]. The production of semiconductor particles with particular schemes is urgent for water splitting to produce hydrogen taking advantage of the visible range of the solar spectrum [17]. A second opportunity (b) lies in further investigation of the deadly disease of septicaemia, through nano-encapsulation of microbes, when antibiotics are not effective. It could be achieved with research using nano-tubular cellulose as a nano-filter or alternatively nano-tubular natural or synthetic polymeric material. Further research is required on these approaches.

NC is the first nano-tubular material characterized as a promoter of a bioconversion (alcoholic fermentation). This creates a new research opportunity (c) to examine this material as a possible promoter of other productive biochemical reactions such as lactic acid fermentation and malolactic fermentation, or the oxidation of alcohol by acetic acid bacteria to acetic acid. Also, its physicochemical structure has been studied in this investigation and could form the basis (d) for synthesis of new catalytic promoters of bioprocesses, having similar polymeric structures. NC can also be employed (e) to produce new composites containing different materials with different properties [18] after introducing inorganic or organic nano-particles in its nano-tubes, as described. In addition, it could be used to strengthen other nano-composites [19]. Nano-tubing in cellulose (f) could also be examined to increase the rate of cellulose hydrolysis by nano-tubes and micro-tubes facilitating the diffusion of cellulases. NC can be employed to accelerate the chemical hydrolysis of cellulose by reversing the technology, from passing cellulose through pores of a resin that contains sulphonic groups [20] to introducing in the tubes of cellulose compounds with sulphonic groups. Furthermore, (g) NC can also be used in the development of novel fermentation methods such as three-phase fermentation, which can be developed by introducing in its tubes non-polar organic solvents that could dissolve the product of fermentation. Therefore, product recovery with lower energy demand could be obtained during distillation, e.g. in fuel-grade alcohol production.

### Conclusions

The produced nano-tubular cellulose (NC) is a suitable new material for multiple applications in bioprocessing. Specifically, it was proved to be effective for removal of microbes in cold pasteurization processes of liquid foods. Also, it has been demonstrated as the first nano-tubular promoter that reduces the activation energy Ea in a bioprocess. Taking into account the chemical and physical structure of NC, the theory of a glucose pump is proposed to explain its promotional activity. The reduction of the activation energy Ea explains the low temperature performance and in combination with the property of NC to aid crystallization of viscous sugar solutions, allows home scale, domestic refrigerator bioprocessing for food production. Such a bioprocess as wine making in a domestic refrigerator was found to improve the quality of the wine produced.

## Materials and Methods

### Ethics Statement

Due to the nature of the research and because no human or animal experimentation was conducted, no ethics statement is required.

### Characterization of the Nano-tubular cellulose structure

The studied nano-tubular cellulose material was produced by delignification of softwood sawdust with NaOH [4]. The average pore diameter and cumulative surface are of pores were measured using a Micromeritics TriStar 3000 porosimeter. For the X-ray studies a ENRAF NONIUS FR 590 X-ray diffractometer was used. The crystallinity [21] and crystallite size [22], [23] of the material was calculated using the X-ray spectra. The scanning electron micrographs were taken using JOEL JSM-6300 and FEI Nova 200 SEM microscopes.

### Cold pasteurization

Cold pasteurization was carried out on aqueous suspensions of *Lactobacillus casei* and the yeast *Saccharomyces cerevisiae* and on liquid orange and apple juices contaminated with *L. casei*. In the case of aqueous microbial suspensions, the experiments were run in a 1.5 L continuous bioreactor system. The cylindrical glass bioreactor (1.5 L) was filled and packed with NC and pumped daily with 1 L microbial suspension of 8 log cfu mL^−1^ at 26°C using a high accuracy peristaltic pump. When the removal fell from 100 to 90%, 5 L of boiling water were pumped through the bioreactor to regenerate the surface of NC. In the case of fruit juices, orange juice contaminated with *L. casei* at 7 log cfu mL^−1^ was used in the same experimental unit. Orange juice was produced by squashing oranges, pasteurization at 62°C for 30 minutes and cold filtration in the laboratory. The pH was adjusted to 3.2 with NaHCO_3_. The bioreactor was fed for 6 days at 3–5°C in a domestic refrigerator. Daily estimations of cell counts at the outlet were performed using the Standard Plate Counting method.

### Solid crystalline grape must production

Thermally dried NC (27.5 g) was mixed with 190 mL grape must of 20° Be density. This mixture was agitated overnight and freeze dried in a Labconco Freezone 4.5 Freeze Dry system. This produced a crystalline mass of grape must.

### Bioconversion in the refrigerator

Cell immobilization of the strain *S. cerevisiae* AXAZ-1 on NC was performed according to a previous study [4]. The solid biocatalyst was mixed separately with solid crystalline grape must, invert sugar and raisins and was freeze dried. Storage of these mixtures for one week at 4°C followed and then tap water was added to obtain the initial sugar concentrations. The mixtures were placed in a refrigerator and fermentation was done at 1°C. Kinetics of bioconversion were measured by analyzing sugar at various time intervals using a Shimadzu HPLC system [24]. Samples were also analyzed for volatile by-products by GC and GC-MS analysis [25]. Sensory evaluation was performed by 14 laboratory members (7 previously trained and 7 untrained) using locally approved protocols. The panel was asked to score the wines based on a 0 to 10 scale (0 unacceptable, 10 exceptional).

### Calculation of activation energy

Fermentations of 400 mL of 12% (w/v) glucose medium were carried out with immobilized and free cells at various temperatures. The activation energies of the fermentation systems were calculated based on the Arrhenius equation according to a previous study [24] by a curve obtained by plotting ln(dP/dt) versus T^−1^.
